# Rethinking HIV prevention in young people: “hierarchization” or “deinstitutionalization”?

**DOI:** 10.1590/0102-311XEN164823

**Published:** 2023-12-08

**Authors:** Draurio Barreira, Tatianna Meireles Dantas de Alencar

**Affiliations:** 1 Departamento de HIV/Aids, Tuberculose, Hepatites Virais e Infecções Sexualmente Transmissíveis, Ministério da Saúde, Brasília, Brasil.

The paper by Grangeiro et al. ^1^ bring to the debate a qualified, contemporary, and timely discussion on the challenges of HIV prevention in young people and on what we mean by “safe sex” after four decades of epidemic. This is a particularly important debate due to the emergence of new preventive technologies in the last decade and a deep sociocultural transformation to the forms of affective, sexual, and substance use interactions by people aged from 15 and 24 years.

Corroborating this scenario, epidemiological studies and data confirm the increase in the detection rate of AIDS cases in this age group (between 2010 and 2021, the detection rate of reported AIDS cases among people between 15 and 24 years ranged from 11.5 to 13.3 ^2^), the trend of lower use of condoms ^3^, and the low number of young people and adolescents among users of HIV pre-exposure prophylaxis (PrEP) in the Brazilian Unified National Health System (SUS) ^4^.

The authors historically revisit the agendas and political actions of LGBTQIAPN+ social movements in the field of HIV prevention and discuss important transformations that took place in the spaces of sociability and, more broadly, the sexual culture of the youth, such as interactions in virtual social media and the practice of chemsex.

The study also brings a core provocation for prevention policies and programs: that simply providing a “*comprehensive package of preventive measures*” and “*equating all methods within a basic package*” have configured limited adoption of safer and more effective practices among the young people and adolescents at higher risk of HIV. Thus, the authors propose that the reorganization of prevention supplies “*necessarily lead to setting a list of priorities to provide prevention methods*” in a focused way, with PrEP being its priority method.

In fact, the diversity of effective methods to prevent HIV has enormously grown in the last decade, questioning the monopoly of condoms and bringing important technological innovations to the scenario, from self-tests to prophylaxis. It could even be said that never before there existed so many preventive methods available in the history of the AIDS epidemic in Brazil and in the world.

It is worth asking whether the issue imposed today for HIV prevention is the lack of hierarchization of the “core package” methods and whether this proposal of hierarchization would lead us to a greater effectiveness of the use of PrEP among young people and the reduction of new cases.

The repositioning of new HIV prevention technologies and the transformations of sexual interactions in the last decade have shown that we should not overvalue a method of prevention, as with condoms in the last 40 years. Today, in the face of so many options, rather than choosing a method of prevention, the issue revolves around prioritizing the innovation of programmatic proposals that provide access to it.

Nor it involves believing in the maxim that the freedom of choice of young people will lead us to control the epidemic. On the contrary, we should recognize, in the context of public health policies, the limits and potentialities of all available preventive methods and consider the complexity of facing the programmatic challenges of HIV prevention.

In this sense, the current HIV prevention policy should prioritize the most vulnerable population in order to expand access and subvert the traditional logic of health care centered solely on healthcare facilities and providers.

After achieving the “technological leap”, we need a “programmatic leap” in the approach, dissemination of information, and the models for providing prevention in a simple, timely and equitable way that dialogues with the needs of users and their age, cultural, social, and economic conditions.

Thus, offering prevention methods at the earliest opportunity, whether via telecare, basic health units, pharmacies, extramural actions, communities, schools, or cultural facilities may be more inclusive than prioritizing methods to expand access.

The hierarchization of prevention methods also fails to guarantee that the needs and socio-cultural dimensions of young people are respected and may even be established only from the perspective of public authorities for their effectiveness, economy, or practicality, which fails to necessarily mirror users’ perspectives.

In addition to locus, the actors involved in expanding access to HIV prevention must go beyond health prescribers. We have learned a lot from peer prevention actions ^5^ and there remains the enormous challenge of “demedicalizing” and “deinstitutionalizing” biomedical prevention strategies with each emerging product. In this time of the epidemic and technological innovations, it is less about the tool and more about the uses we have been able to make of it.

Another essential aspect the study raised refers to promoting messages and approaches for new prevention technologies as ways of having a sexual life with more pleasure, fulfilling desires, and without fear and moral judgments.

In their care, healthcare providers have daily faced adolescents in their first years of their sexual experiences who are paradoxically surrounded by an infodemic ^6^ and by so many prevention technologies available and were recently infected with HIV. Young people and adolescents must have the right to access information and prevention supplies to protect themselves ^7^ in the simplest and closest way to their daily activities, free from moral judgments about the method or their sexual practices.

If the number of PrEP dispensations is still negligible among adolescents from 15 to 18 years of age - since the inclusion of this population as eligible for prophylaxis a little over 11 months ago ^8^ -, this fact can be partially explained by the resistance of healthcare providers to the method ^9^, relevant information that requires address to build a more effective policy that includes strategic priorities that are not necessarily hierarchical.

As Grangeiro et al. ^1^ reiterate, actions to cope with the social dimensions of the HIV epidemic, when combined with prevention strategies, have more effectively reduced new cases. We will fail to advance the provision of prevention strategies, whether “egalitarian” or “hierarchical”, if we continue to be restricted to the field of health and dissociated from a deep social mobilization and intersectoral response.

Reciprocating the good “provocation” the study arouses in us, we ask ourselves if it would not be the case to think of a “hierarchy” that encourages HIV prevention as a priority promoted among peers with the proper support of health services if needed without necessarily being seen as an action inherent to healthcare establishments and providers. One such example could refer to PrEP being initiated by peers.

We have the enormous challenge as public agents of proposing a policy that offers access to prevention strategies for young people and adolescents in an innovative and equitable way; and, as a society, to engender structural changes that contribute to a social representation of AIDS with less stigma, based on human rights and with the leading role of the most affected people.
